# Does Cold Water or Ice Slurry Ingestion During Exercise Elicit a Net Body Cooling Effect in the Heat?

**DOI:** 10.1007/s40279-017-0842-8

**Published:** 2018-01-24

**Authors:** Ollie Jay, Nathan B. Morris

**Affiliations:** 1 Thermal Ergonomics Laboratory, Exercise and Sport Science, Faculty of Health Sciences, University of Sydney, 75 East St., Sydney, NSW 2141 Australia; 2 Charles Perkins Centre, University of Sydney, Sydney, NSW Australia

## Abstract

Cold water or ice slurry ingestion during exercise seems to be an effective and practical means to improve endurance exercise performance in the heat. However, transient reductions in sweating appear to decrease the potential for evaporative heat loss from the skin by a magnitude that at least negates the additional internal heat loss as a cold ingested fluid warms up to equilibrate with body temperature; thus explaining equivalent core temperatures during exercise at a fixed heat production irrespective of the ingested fluid temperature. Internal heat transfer with cold fluid/ice is always 100% efficient; therefore, when a decrement occurs in the efficiency that sweat evaporates from the skin surface (i.e. sweating efficiency), a net cooling effect should begin to develop. Using established relationships between activity, climate and sweating efficiency, the boundary conditions beyond which cold ingested fluids are beneficial in terms of increasing net heat loss can be calculated. These conditions are warmer and more humid for cycling relative to running by virtue of the greater skin surface airflow, which promotes evaporation, for a given metabolic heat production and thus sweat rate. Within these boundary conditions, athletes should ingest fluids at the temperature they find most palatable, which likely varies from athlete to athlete, and therefore best maintain hydration status. The cooling benefits of cold fluid/ice ingestion during exercise are likely disproportionately greater for athletes with physiological disruptions to sweating, such as those with a spinal cord injury or burn injuries, as their capacity for skin surface evaporative heat loss is much lower; however, more research examining these groups is needed.

## Introduction

During prolonged aerobic exercise, metabolic rate rises substantially from rest to provide the body with the energy needed to perform large amounts of muscular work [1]. Subsequently, the vast majority (> 70 to 75%) of metabolic energy is liberated as heat and must be dissipated from the skin to the surrounding environment to maintain internal body (core) temperature within optimal limits [2]. Ultimately, if skin surface heat loss, which is physiologically mediated by sweating and cutaneous vasodilation responses but also greatly dependent upon environmental conditions, is insufficient, the prevailing large elevations in core and skin temperature contribute to profound decrements in endurance sports performance [3]. For example, time to exhaustion during exercise at a fixed intensity can be more than halved at an ambient air temperature of 31 °C compared with 11 °C [4], while self-paced marathon completion time increases by ~ 12% at a wet bulb globe temperature of 25 °C relative to 10 °C [5].

In an attempt to mitigate heat-related impairments in aerobic exercise, performance athletes and sports practitioners regularly employ different cooling strategies both before (pre-cooling) and during (mid-cooling) exercise [6, 7]. Research has shown that pre-cooling athletes, using cold water immersion (2–20 °C) [8, 9], ice vests applied to the torso [10] or neck cooling collars [11, 12] blunts heat-related decrements in performance [7]. While likely beneficial, most of these interventions are not particularly feasible in low-resource environments (such as away-games, competition in remote areas and amateur sport) and have limited application as a cooling strategy during exercise, especially competition. Arguably, the most practical cooling solution is the ingestion of cold water or an ice slurry (cold water mixed with crushed ice) before and/or during exercise.

The evidence supporting the efficacy of ice slurry or cold (< 10 °C) water ingestion for improving endurance exercise performance in the heat when employed as a pre-cooling [13–21], mid-cooling [22–28] or recovery between bouts [29, 30] strategy has been comprehensively reported. However, whether these strategies actually reduce the amount of heat stored inside the body during exercise in the heat, even at a fixed metabolic rate, is currently unclear. The underlying rationale for using cold water or ice slurry ingestion is that additional internal heat transfer is introduced as the ingested bolus heats up to equilibrate with internal body temperature. The total amount of heat transfer is dependent upon the temperature difference between the ingested fluid and the body, the specific heat capacity of the fluid (4.186 kJ·kg^−1^·°C^−1^ for water) and the volume/mass ingested. Moreover, the ingestion of an ice slurry mixture induces even more heat transfer owing to the enthalpy of fusion of ice (334 kJ/kg). Nevertheless, this internal heat transfer occurs as part of a dynamic system of whole-body heat balance that is profoundly altered by physiological responses (primarily sweating) and the characteristics of the environment that envelops the athlete (i.e. air temperature, humidity, wind speed and radiant heat load). As such, how successfully cold water or ice slurry ingestion ultimately impacts whole-body heat content during exercise in the heat is greatly dependent on the parallel sweating response and the prevailing environmental conditions.

The aims of this review are to (1) describe the physiological and biophysical factors determining whether a cold fluid or ice slurry ingestion results in a net cooling effect from a human heat balance perspective; and (2) identify the boundary environmental conditions at which cold fluid or ice slurry ingestion should be recommended as an effective strategy for reducing body heat content during exercise.

## Fundamentals of Human Heat Balance during Athletic Competition

The extent to which an athlete’s body temperature changes during exercise is determined by (1) the net difference between internal heat generation and skin surface heat dissipation (body heat storage), (2) body mass and (3) specific heat capacity of the body’s tissues [2]. As the body mass and specific heat capacity of an athlete do not change dramatically across the course of a typical sporting event (except in extreme cases, e.g. Marathon des Sables [31]), cooling interventions can solely serve to alter an athlete’s body temperature by modifying body heat storage, which is classically described using the conceptual human heat balance equation [32]:

1 $$ S = \left( {M - W} \right) \pm K \pm C \pm R - E. $$

Metabolic heat production is determined by metabolic energy expenditure (*M*) minus the amount of external work (*W*) that is concomitantly produced. Metabolic energy expenditure is directly governed by the athlete’s absolute rate of oxygen consumption (*V*O_2_; in L·min^−1^), and the proportion of this oxygen that is used to catabolise carbohydrates (yielding 21.13 kJ of energy per 1 L of O_2_ consumed) relative to fats (yielding 19.69 kJ of energy per 1 L of O_2_ consumed). The rate of external work performed on a bicycle is chiefly determined by pedalling speed, and the external resistance that must be overcome, which itself is modified by the weight of the rider, the gradient and the type of terrain. Because of continuous friction between tyres and the ground, external work must still be performed even when cycling on a flat surface. Running on a flat surface in contrast results in a negligible amount of external work as the propulsion and breaking forces of gait result in equal amounts of positive and negative work, respectively [33, 34]. The amount of external work performed by running up a hill is solely determined by the vertical displacement of the athlete [34], but remains relatively small (< 5% of M) compared with cycling (~ 20 to 25% of M).

The net rate of heat loss from the skin surface to the surrounding environment is the sum of heat exchange via conduction (*K*), convection (*C*), radiation (*R*) and evaporation (*E*). Conduction, which is heat transfer through direct contact with a solid surface, is generally considered negligible for most exercise scenarios [35]. For a runner, as an example, the only direct physical contact with a solid surface occurs between the soles of the feet, which have a relatively small contact area and are usually insulated by shoes, and the ground.

Convective heat exchange is accelerated by the flow of air moving across the skin surface, which can arise from environmental air movement and/or self-generated air movement as an athlete propels themselves through an air mass. Convection is also directly dependent upon the temperature difference between the skin and the ambient environment. When air temperature is equal to skin temperature (~ 33 to 35 °C), no convective heat exchange occurs irrespective of air velocity. At even warmer air temperatures (> 35 °C), convective heat loss becomes convective heat gain, that is, dry heat is added to (as opposed to removed from) the body, which is further accelerated with higher air velocities [36, 37]. This phenomenon is analogous to the faster cooking time of food in a fan-assisted oven compared with a conventional oven. It is also worth noting that additional convective heat exchange also occurs via respiration but in terms of whole-body heat exchange the contribution is relatively small, especially in warm environments [38].

Radiative heat exchange is electromagnetic energy transfer between a relatively warm and cool body. Similarly to convection, *R* is driven by the temperature gradient between the skin and the environment, however, in this case mean radiant temperature—determined using black globe temperature and air velocity—is the important environmental parameter. In hot and particularly sunny climates, thermal radiation is added to the body as a result of the higher mean radiant temperature relative to skin temperature. The greatest source of thermal radiation in most sports-related settings is almost always solar radiation. Depending on the time of day, season, cloud cover and latitude, black globe temperature can be as much as ~ 15 °C higher than ambient air temperature. For example, on a hot and sunny mid-summer day in Sydney, Australia, black globe temperature at 1 pm can be as high as 50 °C when ambient air temperature (measured in the shade) is ~ 35 °C [39]. The posture and orientation of the athlete relative to the sun, which determine the effective radiative area of the body, will also substantially contribute to the amount of thermal radiation absorbed by the body [35].

In humans, heat loss by evaporation occurs as a result of a difference in humidity between the skin surface, which is physiologically moistened by eccrine sweating, and ambient air. It is worth emphasising that absolute humidity is the critical environmental factor influencing evaporation, and not relative humidity (RH), which is the most commonly provided metric in meteorological reports. Absolute humidity at a fixed RH increases exponentially with ambient temperature, described by Antoine’s equation [35], to the extent that twice the amount of moisture is present at 30%RH/38 °C than at 30%RH/26 °C. Similarly, absolute humidity is higher at 30%RH/40 °C than at 50%RH/30 °C. In circumstances where all sweat does not readily evaporate and residual sweat sits on the skin (e.g. exercise coupled with high humidity), sweat evaporation is also heavily influenced by airflow across the skin surface. That is, evaporative heat loss is much higher in a hot, dry and windy environment compared with a hot, humid and still environment.

Humans have an upper limit for evaporative heat loss (*E*_max_) that is determined by different characteristics depending upon the type of environment. In hot but very dry environments, *E*_max_ is capped by the physiological capacity to secrete sweat [3]; but in more humid environments, *E*_max_ is determined by the maximum proportion of total body surface area that can be completely covered with sweat, i.e. maximum ‘skin wettedness’ (*ω*_max_) [40]. Values for *ω*_max_ range from 1.00, which indicates that the entire body surface area is completely covered with sweat and is only observed in fully heat-acclimated athletes [41], through to 0.80–0.85 in non-heat-acclimated persons [41].

Recent data [42] from our laboratory indicate that the partial heat acclimation associated with 8 weeks of aerobic training in a temperate environment leads to *ω*_max_ values of ~ 0.90 to 0.95. However, *ω*_max_ values in athletes with injuries that have greatly altered regional sweat gland function, e.g. spinal cord-injured athletes or athletes with burn injuries, will only be able to achieve much lower whole-body *ω*_max_ values, depending on the extent of injury (e.g. size and region of area burned; level and completeness of spinal cord injury). However local skin wettedness on the skin areas that can sweat will reach higher values for a given whole-body sweat rate. Irrespective of acclimation or training status, as skin wettedness increases towards its maximum value, sweating efficiency, i.e. the amount of sweat that evaporates relative to the amount of sweat produced, drastically decreases [43]. Sweating efficiency is an important notion because it is the evaporation of sweat that liberates heat from the body (2430 J of latent heat for every 1 g of sweat vaporised [35]) and not the production of sweat. It follows that under conditions that yield a low sweating efficiency, a reduction in sweat rate may not necessarily impact evaporative heat loss from the skin if that sweat would otherwise just remain on the skin or drip off the body.

**Fig. 1:**
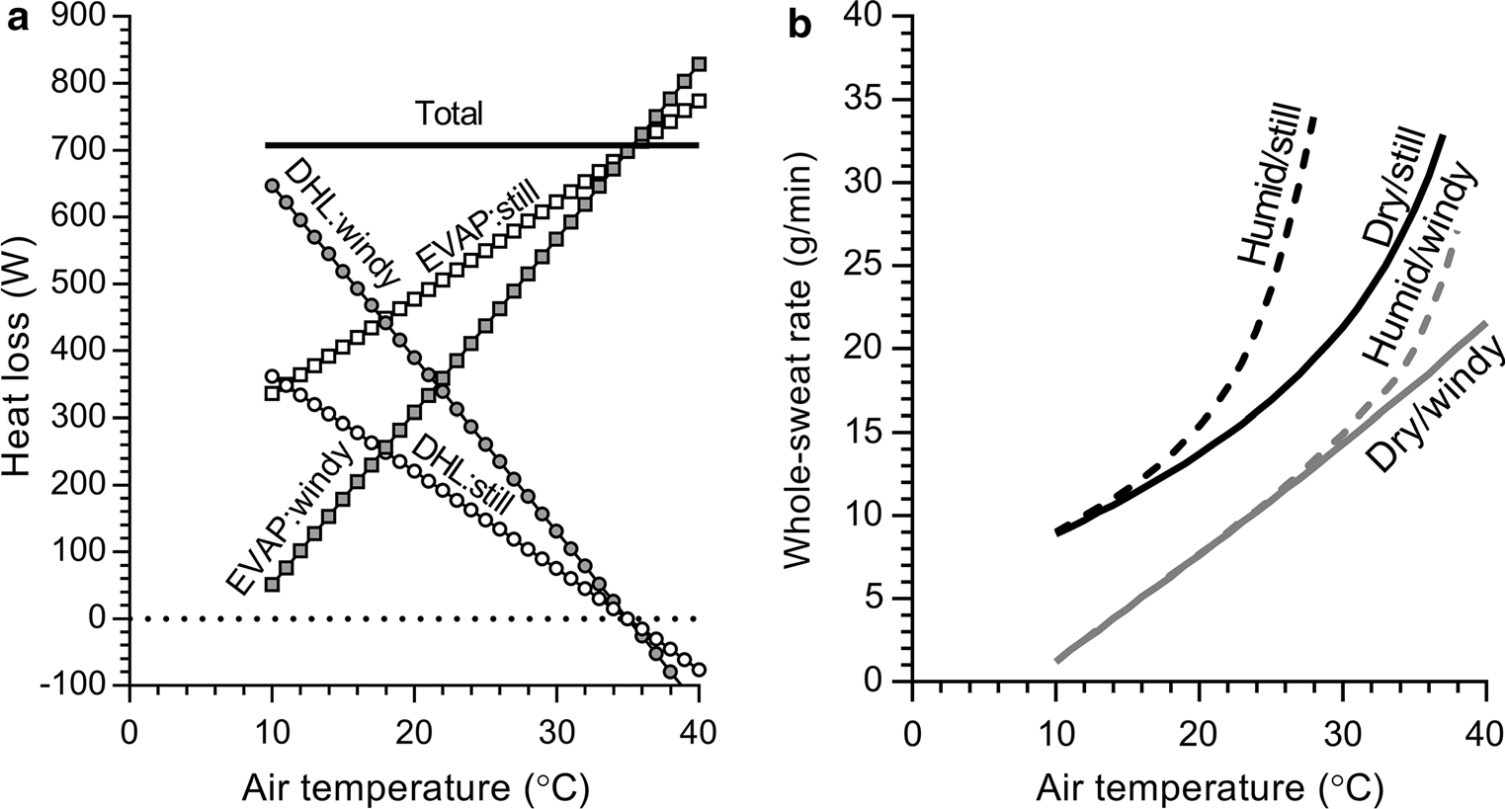
Changes in dry heat loss [DHL = conduction (*K*) + convection (*C*) + radiation (*R*)] and the required evaporation (EVAP) to achieve a total heat loss (total HL) of 700 W with increasing air temperature under still [air velocity (*v*): 0.5 m·s^−1^] and windy (*v*: 5.0 m·s^−1^) conditions and no solar load (**a**). Parallel whole-body sweat rates required to achieve the levels of EVAP depicted in (**a**) are shown in (**b**) for dry [relative humidity (RH) 25%] and humid (RH 60%) conditions

The change in body heat storage (*S*) during exercise is determined by the cumulative difference in metabolic heat production (*M* − *W*) and net heat dissipation from skin to the surrounding environment (± *K* ± *C* ± *R* ± *E*) [32]. During exercise at a fixed intensity (and thus *M* − *W*), which requires a fixed rate of net heat dissipation to achieve heat balance as described in Eq. 1, the amount of evaporative heat loss (*E*) required increases as the amount of dry heat loss (*K* + *C* + *R*) decreases with a progressively warmer environment (Fig. 1). The rate of whole-body sweating required to achieve the required rate of evaporation is altered by sweating efficiency, which itself is determined at a fixed exercise intensity by a combination of ambient humidity and air flow across the skin of the athlete (Fig. 1). When ambient air temperature exceeds skin temperature, which is typically ~ 35 °C, all skin surface heat dissipation must occur via the evaporation of sweat. It follows that in a hot, still and humid environment, an athlete is unable to physiologically compensate metabolic heat production with sufficient evaporation. Indeed, under such conditions additional sweat secretion does not serve to enhance heat loss as it is destined to simply drip off the body and not contribute to evaporation [41]. The subsequent development of ‘uncompensable’ heat stress, which is most prominently characterised by a sustained rate of rise in core (and usually skin) temperatures [44] owing to a continuous rate of heat accumulation inside the body, greatly increases the risk of heat-related illness and injury for an athlete [45].

## How Does Cold Fluid or Ice Slurry Ingestion Impact Human Heat Balance?

**Fig. 2:**
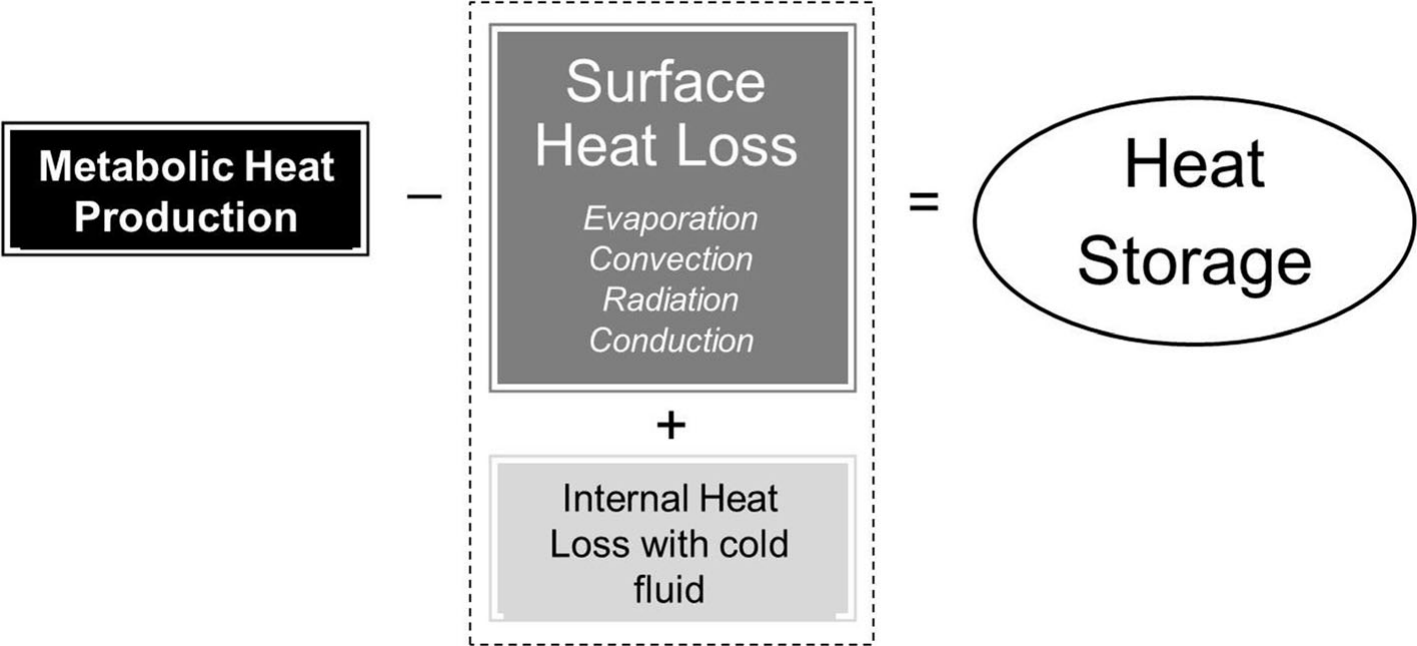
Determinants of body heat storage with cold fluid ingestion during exercise

Cold fluid ingestion introduces a new avenue of heat transfer (internal heat transfer) for an athlete exercising in the heat in addition to the four avenues of heat transfer at the skin surface [14, 25, 46]. Body heat storage with cold fluid ingestion is therefore determined by the cumulative difference between metabolic heat production and the combined heat loss from the skin surface and any internal heat transfer with an ingested fluid (Fig. 2). It stands to reason that if net heat dissipation from the skin remains unaltered with cold fluid ingestion, body heat storage will be lower and the athlete will likely be at a lower risk of heat-related illness. Given that most skin surface heat loss during exercise in hot climates must occur via evaporation, any physiological modifications of sweating with cold fluid ingestion will have the greatest impact on heat balance. Moreover, if any reductions in skin blood flow also occur with cold fluid ingestion, then any consequent changes in skin temperature may also modify the rate of dry heat loss via convection and radiation occurring at the skin surface. Ultimately, if eccrine sweating and skin blood flow are reduced with cold fluid ingestion to the extent that skin surface evaporation and dry heat loss are lowered in proportion to the internal heat transfer taking place mostly inside the stomach, then combined heat loss (i.e. skin surface + internal) may not be enhanced relative to the ingestion of a thermoneutral fluid.

### Modifications of Physiological Heat Loss Responses with Cold Fluid/Ice Slurry Ingestion during Exercise and Their Impact on Heat Balance

The influence of cold fluid ingestion on sweating and heat balance was first described in resting individuals in a 1942 study by Pinson and Adolph [47]. They reported that ingestion of 1.4–1.7 L of 1–3 °C water at rest in a 31 °C and 20–30% RH room led to a ~ 50% decrease in evaporative heat loss from the skin, with losses remaining depressed for 3 h post-ingestion. Through partitional calorimetry they reported that 200 min after ingestion, 85% of the internal heat loss to the ingested fluid had been regained; half by decreases in skin surface evaporation and half by decreases in dry heat loss via convection, suggesting an effect of cold fluids on both sweating and vasodilation. Later, Nadel and colleagues [48] characterised the effect of ingesting two different masses of ice cream (518 and 260 g) and different temperatures of hot pudding (37 °C and 55–60 °C) in participants sitting in environments ranging from 10 to 44 °C. In the coolest condition (10 °C), ice cream ingestion caused a marked increase in metabolic heat production and thus shivering thermogenesis, whereas in the hottest condition (44 °C), ice cream ingestion decreased sweating and skin blood flow, and hot pudding ingestion caused marked increases in sweating and vasodilation.

Wimer et al. [49] were the first to examine sweating responses of participants drinking fluids of different temperatures during exercise (2 h of cycling at ~ 50% *V*O_2peak_). They reported whole-body sweat losses of 471 g·h^−1^ with 0.5 °C water ingestion compared with 679 g·h^−1^ with 38 °C water ingestion of the same volume, as well as parallel changes in blood flow and local sweat rate on the forearm. More recently, our research group reported progressively lower whole-body sweat losses with declining ingested water temperature after 75 min of exercise at 50% *V*O_2max_ in a 24 °C environment. Sweat losses ranged from 575 g with the ingestion of ~ 1 L of 50 °C water to 465 g with the ingestion of the same volume of 1.5 °C water [50]. From a heat balance perspective, compared with the ingestion of a thermoneutral (37 °C) fluid, the ~ 140 kJ of additional internal heat loss in the 1.5 °C water trial was counterbalanced by an almost proportional ~ 160 kJ reduction in potential sweat evaporation from the skin. Similarly, the ~ 100 kJ internal heat loss with the ingestion of 10 °C water was balanced by a ~ 105 kJ reduction in evaporative potential from the skin.

To perform further analyses of alterations in human heat balance with cold water ingestion during exercise, the existing literature was searched to capture all studies reporting whole-body sweat losses (to enable the estimation of changes in evaporative potential) following the ingestion of water of at least two different temperatures of a known volume (to enable the estimation of internal heat losses) during steady-state exercise in a non-encapsulated environment. Exercise performance studies were excluded as they employ variable exercise intensities and thus rates of metabolic heat production, which itself would alter sweating independently of ingested fluid temperature [22]. Additionally, studies that did not report whole-body sweat loss values were also excluded as evaporative heat loss could not be estimated [51, 52]. In total, ten studies were included (Table 1), with only three reporting responses with ice slurry ingestion because all other ice slurry studies we found in the literature examined changes in exercise performance. The range of ingested water temperatures represented by the other seven captured studies was from 0.5 to 50 °C (Table 1).

**Table 1:** Studies reporting sweat losses and core temperature (*T*_C_) responses during steady-state (fixed-intensity) exercise over a fixed time with ingestion of at least two water temperatures, or one water temperature compared with ice slurry ingestion

References, year	*n*	*T*_a_ (°C)	RH (%)	*T*_fluid_ (°C)	End-trial *T*_C_ (°C)	Volume (mL)	*H*_fluid_^a^ (kJ)	Sweat loss (g)	*E*^b^ (kJ)
Gisolfi and Copping, 1974 [78]	6	33.5	36	10, 38	39.2, 39.4	1200	147, 1	3124, 3154	7591, 7664
Wimer et al., 1997 [49]	7	26	40	0.5, 19, 38	38.0, 38.0, 38.1	1353	212, 108, 1	471, 551, 649	1144, 1339, 1571
Lee and Shirreffs, 2007 [25]	9	25.4	61	10, 37, 50	38.2, 38.2, 38.3	1000	118, 5, − 49	1230, 1260, 1320	2989, 3062, 3208
Lee et al., 2008 [26]	8	25	60	10, 37, 50	38.1, 38.1, 38.2	1200	141, 6, − 59	1090, 1230, 1350	2649, 2989, 3281
Bain et al., 2012 [50]	9	23.6	23	1.5, 10, 37, 50	38.0, 37.9, 38.0, 38.1	1030	141, 104, 3, − 49	465, 488, 531, 575	1129, 1184, 1288, 1396
Morris et al., 2014 [55]	8	23.7	32	1.5, 50	37.4, 37.4	705	106, − 37	630, 745	1530, 1810
Lamarche et al., 2015 [53]	10	25.9	25	1.5, 50	38.0, 37.9	1014	150, − 54	560, 634	1358, 1538
Burdon et al., 2013 [23]	10	32.1	40	Ice, 37	38.2, 38.3	1560	600, 8	500, 600	1215, 1458
Hailes et al., 2016 [24]	12	35.5	50	Ice, 35.5	38.1, 38.2	1330, 2660	511, 30	2517, 2703	6116, 6568
Morris et al., 2016 [46]	9	33.5	24	Ice, 37	37.7, 37.7	729	201, 1	568, 720	1829, 2211

*RH* relative humidity, *T*_*a*_ ambient temperature

^a^Internal heat transfer (*H*_fluid_) calculated using fluid volume, the specific heat capacity of water (4.186 J·g^−1^·°C^−1^), the enthalpy of fusion of ice (334 J/g) if required and the difference between fluid temperature (*T*_fluid_) and *T*_C_

^b^Evaporative potential from the skin (*E*) calculated using sweat loss, the latent heat of vaporisation of sweat (2430 J/g) and assuming 100% evaporation

**Fig. 3:**
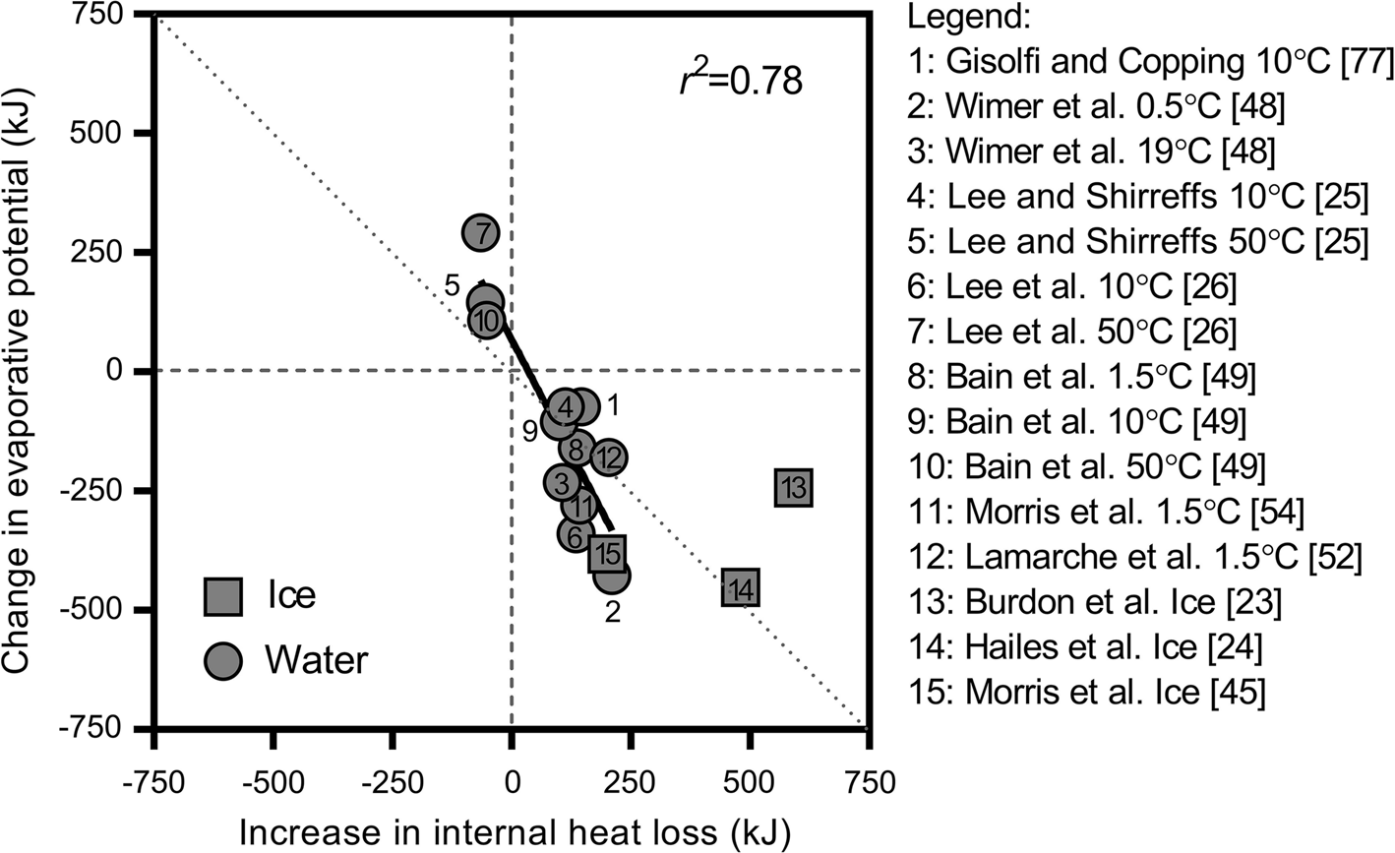
Association between changes in evaporative potential from the skin surface with changes in internal heat loss with ingested fluid/ice. Dotted line indicates line of identity. A best fit line is provided for water ingestion trials only

We estimated differences in evaporative heat loss potential from the skin relative to a control fluid temperature using differences in whole-body sweat losses and the latent heat of vaporisation of sweat (2430 J·g^−1^; [35]). We plotted these values (Fig. 3) against parallel differences in internal exchange estimated using fluid volumes administered throughout exercise, change in fluid temperature (core temperature−ingested fluid temperature) and the specific heat capacity of water (4.186 J·g^−1^·°C^−1^) and, if required, the enthalpy of fusion of ice (334 J·g^−1^). A strong negative association is evident, that is, as internal heat loss increases with the ingestion of progressively colder fluids, a parallel decrease in evaporative potential from the skin occurs. Indeed, this observation was true for all studies—evidenced by all captured cold water data points falling within the bottom-right quadrant (Fig. 3).

Similarly, the three data points that assessed the relative influence of an internal heat load with the ingestion of a warm fluid resulted in an increase in evaporative potential from the skin supported by elevated sweat rates (top-left quadrant of Fig. 3). As the best-fit regression line for the association does not fall directly along the line of identity, the data indicate that alterations in evaporative potential may not be in direct proportion to changes in internal heat transfer (Fig. 3). That is, reductions in evaporative potential seem to be greater than the increase in internal heat loss with cold water ingestion, and the increase in evaporative potential appears greater than the relatively small amount of internal heat added to the body with warm water ingestion.

This observation however must be viewed with an understanding that methodological approaches for measuring sweat rate differed substantially between studies and the environmental conditions and exercise intensities selected may have induced varying levels of sweating efficiency [41, 43]. Indeed, the additional sweat in the warm fluid conditions may not have actually evaporated; however, in the cool fluid studies/conditions, variations in sweating efficiency do not explain why the reduction in evaporative potential is often disproportionately greater. Moreover, some of the studies included in our analysis did not report significantly different whole-body sweat losses between fluid temperatures, possibly owing to a combination of relatively small sample sizes and the precision of measurement. Nevertheless, we used mean values reported by all studies.

Lamarche et al. [53] probably provide the most reliable data because they attained the most precise estimates of human heat balance by employing whole-body direct calorimetry. They reported that the ~ 205 kJ greater internal heat loss with a 1.5 °C compared with a 50 °C water drink was almost fully negated by a ~ 180 kJ reduction in sweat evaporation, with the remaining ~ 25 kJ of internal heat loss cancelled by a similar reduction in dry heat loss [53]. Their data also indicate that the disproportionately greater evaporative potential with warm fluid ingestion observed by Bain et al. [50], Lee et al. [25] and Lee et al. [26] may have been a function of a reduction in sweating efficiency. Alterations in human heat balance with ice slurry ingestion appear to diverge substantially among the three studies captured by the present analysis. A proportional reduction in evaporative potential [24], as well as disproportionate reduction [46], and a disproportionate maintenance [23] of evaporative potential were all reported.

**Fig. 4:**
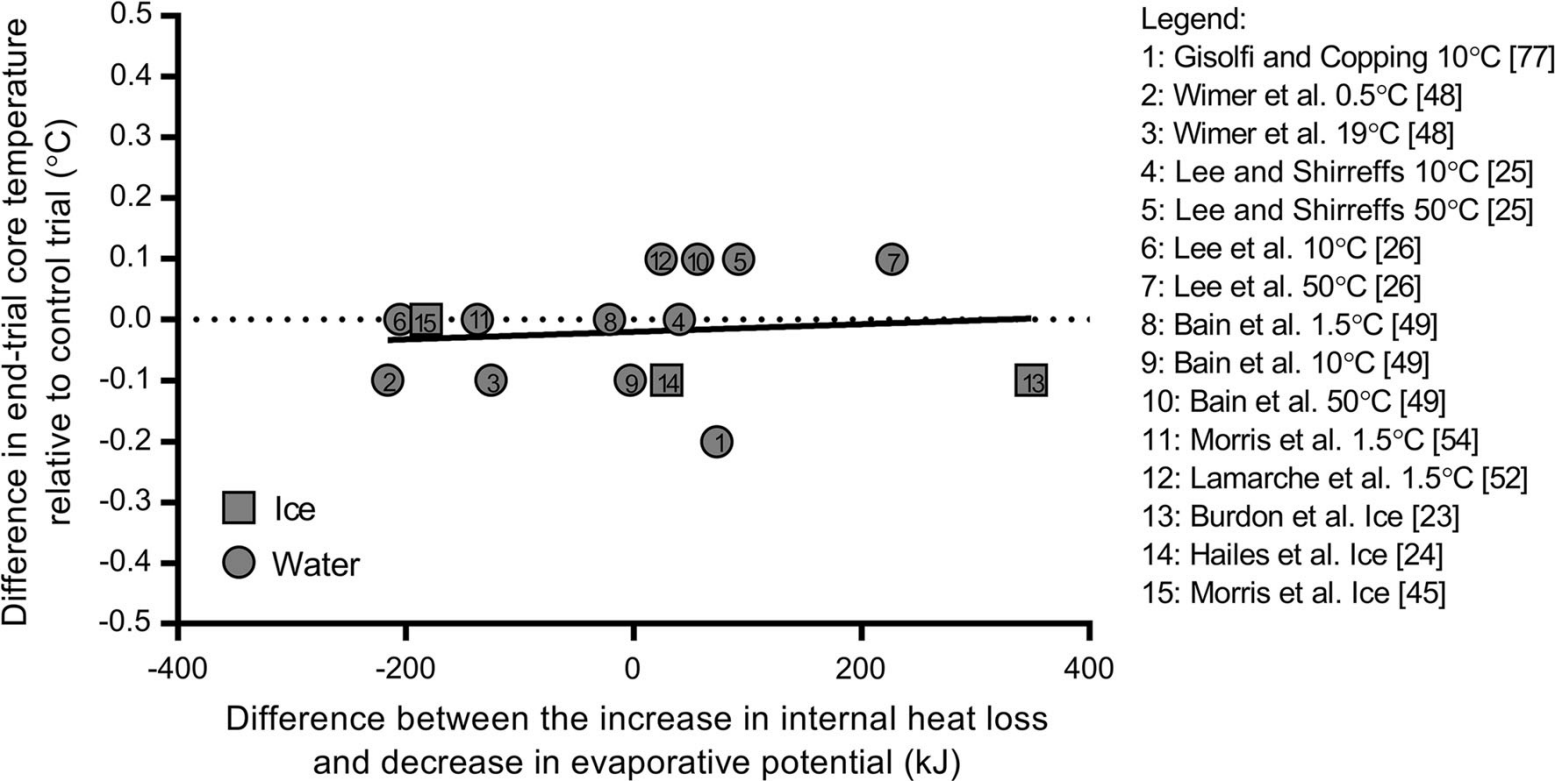
Association between the differences in end-trial core temperature relative to control fluid conditions within each study with net differences between internal heat loss and evaporative potential with ingested fluid/ice. A best-fit line is provided for water ingestion trials only

Irrespective of whether changes in evaporative potential with alterations in internal heat transfer with cold water/ice ingestion are proportional or not, a striking similarity among all captured studies is that at a fixed metabolic heat production any disturbances of heat balance do not deviate sufficiently from the line of identity in Fig. 3 to yield higher or lower core temperatures. Figure 4 illustrates differences in core temperature for all comparisons in Fig. 3 as a function of the difference in internal heat loss and change in evaporative potential. This observation is an essential consideration for athletes exercising in the heat as while they may feel cooler with cold fluid ingestion [23, 28], the data indicate that they will not actually be cooler.

It is worth noting that maximum exercise duration among all captured studies was 180 min [24] and that a longer duration may be required to observe differences in core temperature that arise from an imbalance between internal heat loss and changes in skin surface heat dissipation. Indeed, Senay [54] reported a greater rise in rectal temperature after 12 h of resting exposure to a 43 °C environment in participants drinking a fixed volume of cold (8–10 °C) 0.1% saline every hour, compared with the same volume and rate of ingestion of thermoneutral (37 °C) 0.1% saline. Interestingly, reported reductions in evaporative potential in this study, albeit with only three participants, were three to four times greater than the greater internal heat loss with cold saline intake [54].

### How are Different Sweat Rates Observed Between Fluid Temperatures Without Differences in Core Temperature?

Rapid but transient fluid temperature-dependent changes in local sweat rates have been observed on the forehead, upper back and arm within 1 min of cold and warm water [55] as well as ice slurry [46] ingestion during exercise. It is well understood that hypothalamic control of sweating is primarily determined by afferent information provided by thermoreceptors in the body core, and to a lesser extent the body shell (i.e. skin) [56–58]. Therefore, the fact that vast differences in skin surface evaporative potential secondary to lower sweat rates are observed alongside similar core (and skin) temperatures implies a modifier of sudomotor control that is sensitive to ingested fluid temperature, and not the action of drinking per se, as changes in sweating are not observed with thermoneutral (37 °C) fluid ingestion [46, 55].

A recent study from our laboratory demonstrated that the thermoreceptors responsible most likely reside in, or around, the stomach; mouth-swilling water at 50 and 1.5 °C had no effect on local sweat rates, whereas directly administering 50 and 1.5 °C water to the stomach using a nasogatric tube (which bypassed the mouth) elicited alterations similar to the water temperature-dependent changes in local sweating with regular drinking [55]. We have since expanded these findings by demonstrating that, similar to cold fluid ingestion, ice slurry ingestion decreases local sweat rate and skin blood flow, but to a greater extent [46]. Most recently, we have also reported an independent influence of cold and warm water ingestion on shivering during cold stress [59].

### Under What Circumstances Does Cold Water/Ice Slurry Ingestion Reduce Body Heat Storage?

#### Hot/Humid Environments

**Fig. 5:**
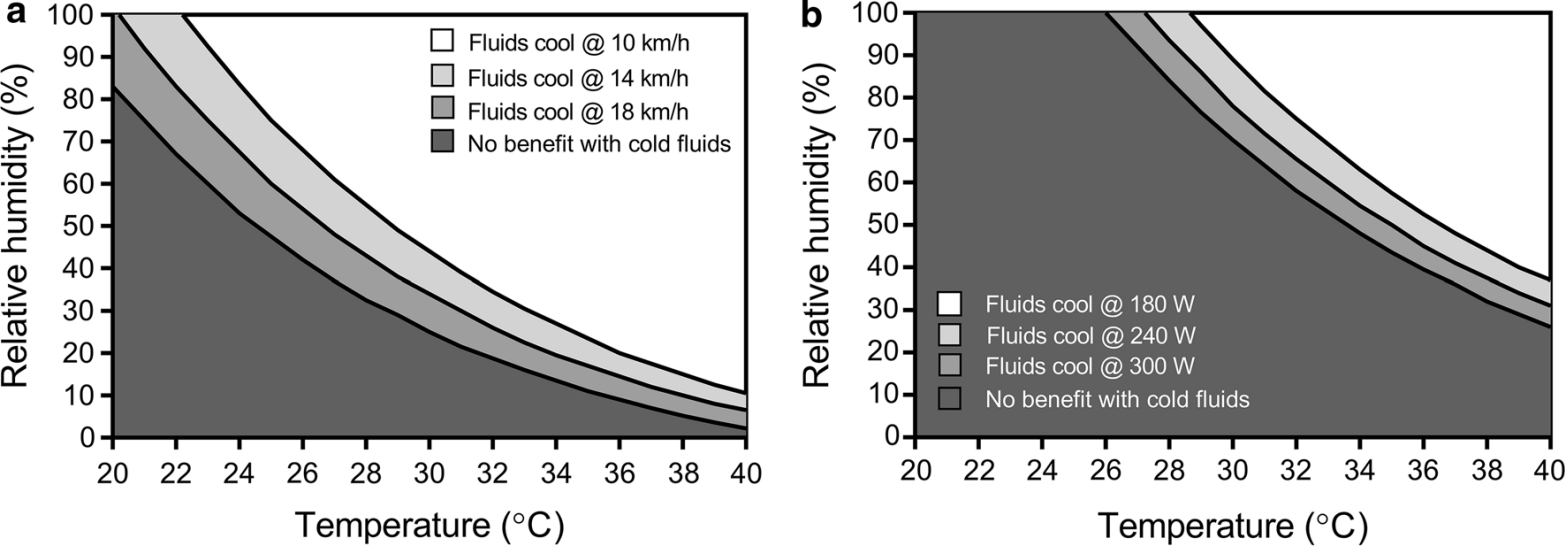
Estimated environmental boundary conditions at which cold water and ice slurry ingestion provides a net cooling effect for running at 10, 14 and 18 km/h (**a**) and cycling on a flat surface at an external power of 180, 240 and 300 W (**b**). Rates of metabolic heat production at different running speeds and cycling power outputs were estimated using standard American College of Sports Medicine equations [79]. Maximum skin wettedness at high air speeds was equal to 0.30 [80]. Boundaries were estimated assuming equivalent reductions in the evaporative heat loss potential from the skin with increases in internal heat loss with cold water/ice ingestion

The primary advantage of inducing heat loss internally with cold water or ice slurry ingestion is that heat transfer is guaranteed to be 100% efficient. In contrast, latent heat dissipation via sweat evaporation is subject to a large variation in efficiency depending on ambient humidity, air velocity across the skin and required sweat rate (dependent upon metabolic heat production). As such, in hot, humid and still climates, reductions in evaporative potential may not result in reductions in actual evaporation; in which case cold water/ice slurry ingestion will be beneficial for reducing body heat storage. It follows that the boundary combinations of air temperature and humidity for different activities (and the accompanying metabolic heat production) at which cold water and ice slurry drinks are beneficial for eliciting a lower heat storage can be estimated. The critical RH above which cold fluids should produce a net cooling effect becomes lower as ambient air temperature increases both for running (Fig. 5a) and cycling (Fig. 5b).

Indeed, in hot and humid conditions, the reduction in sweat output with cold water/ice ingestion does not impact evaporation because additional sweat would only drip off the skin and the maximum evaporation possible is attained anyway. Despite a lower air velocity across the skin, a slower runner, notwithstanding differences in running economy, will generate much less heat and therefore much less sweat will be secreted onto the skin resulting in a higher sweating efficiency—under such a scenario, reductions in sweat output would only compromise evaporative heat loss from the skin at higher air temperatures and humidity (Fig. 5a). As a runner travels faster, they must exercise at a higher metabolic heat production, which would result in high sweat rates with a subsequently low proportion of the sweat actually evaporating from the skin; indeed, evaporative efficiency of sweat in heat-acclimated elite athletes has been reported to be well below 50% [60]. As such, the ambient temperature and RH at which cold fluids would be beneficial in terms of cooling would be drier and cooler for a faster runner (Fig. 5a). For cyclists, ambient airflow across the skin is much higher for a given metabolic heat production when compared with runners, except in the case of steep hill climbing [61]. A high sweating efficiency is therefore maintained at higher air temperatures and humidity for cycling and therefore the boundary conditions for a net cooling effect from cold fluid/ice ingestion are hotter and more humid (Fig. 5b).

#### Pre-Cooling

In contrast to the studies reported earlier in Table 1, in which fluids were administered during exercise, Table 2 contains an aggregate of study findings in which fluids were administered prior to the onset of exercise as a pre-cooling intervention. Here, it is clear that cold fluid or ice ingestion consistently decreases core temperature (by ~ 0.5 °C) before exercise starts. Given that cold fluid ingestion does not seem to be effective for producing a net cooling effect during exercise because of a compensatory reduction in sweat output and subsequently the evaporative potential from the skin, it stands to reason that if cold fluids are ingested before a full sweating response is developed a reduction in body heat storage will be achieved. Indeed, if the resting core temperature remains within the inter-threshold zone, it is defended by neither sweating nor shivering and is allowed to drift with alterations in vasomotor activity solely responsible for slowing the pace of the core temperature change [62, 63].

**Table 2:** Aggregate of studies using cold water or ice ingestion as a pre-cooling intervention prior to an exercise performance trial

References, year	*n*	*T*_a_ (°C)	RH (%)	*T*_fluid_ (°C)	*T*_C_ at rest (°C)	*T*_C_ post-cooling (°C)	End-trial *T*_C_ (°C)	Δ*T*_C_/time (°C·min^−1^)	Volume (mL)	Sweat rate (g·h^−1^)
Lee et al., 2008 [21]	8	35	60	4, 37	36.9, 36.9	36.4, 36.8	39.5, 39.5	0.049, 0.052	300	1220, 1400
Byrne et al., 2011 [13]	7	32	60	2, 37	37.3, 37.3	36.9, 37.1	38.2, 38.6	0.043, 0.050	900	770, 980
Siegel et al., 2010 [14]	10	34	55	Ice, 4	37.2, 37.1	36.5, 36.8	39.4, 39.1	0.058, 0.057	599	1890, 2050
Siegel et al., 2012 [15]	8	34	52	Ice, 37	37.1, 37.1	36.7, 37.1	39.8, 39.5	0.059, 0.051	586	2060, 2280
Yeo et al., 2012 [16]	12	28	75	Ice, 31	37.5, 37.4	37.0, 37.3	40.2, 39.8	0.071, 0.055	511	1130, 1110
Naito and Ogaki, 2015 [18]	9	35	30	Ice, 4	37.1, 37.2	36.8, 37.1	38.9, 38.9	0.042, 0.043	768	1800, 1700
Gerrett et al., 2016 [17]	12	31	41	Ice, 23	37.3, 37.3	36.7, 37.3	38.5, 38.9	0.058, 0.052	551	500, 510
Stevens et al., 2016 [20]	11	33	46	Ice, 22	37.2, 37.2	36.9, 37.2	39.0, 39.1	0.080, 0.073	548	NA
Naito et al., 2017 [19]	7	35	30	Ice, 4	37.1, 37.1, 37.1	36.6, 36.7, 37.0	38.8, 38.7, 38.6	0.048, 0.052, 0.050	530	820, 1200, 720

*NA* not applicable, *RH* relative humidity, *T*_*a*_ ambient temperature, *T*_*c*_ core temperature, *T*_*fluid*_ fluid temperature

One important caveat of using cold water/ice ingestion as a pre-coolant is that the lower core temperature upon the commencement of exercise most likely delays the onset of sweating, and possibly the vasodilatory response, resulting in a greater rate of heat storage and a greater rate of core temperature rise during the early stages of exercise, as has been demonstrated with whole-body forms of pre-cooling [64]. The data in Table 2 seem to support this proposition as the rate of increase in the core temperature is typically greater with ice slurry ingestion compared with the control fluid (Yeo et al. [16], + 0.016 °C·min^−1^; Siegel et al. [15], + 0.008 °C·min^−1^; Stevens et al. [20], + 0.007 °C·min^−1^) but not always greater (Byrne et al. [13], − 0.007 °C·min^−1^).

#### Recovery

While the conditions under which cold fluid/ice ingestion would be beneficial during exercise have been modelled and described (Fig. 5a, b), drinking cold fluids directly after exercise during the early stages of recovery may also successfully create a large net cooling effect. A rapid post-exercise decline in sweating and skin blood flow secondary to non-thermal control inputs associated with baroreceptors, mechanoreceptors and metaboreceptors has been well characterised [65] with a ~ 50% reduction in sweat output observed within 5 min of stopping exercise despite little (or no) reduction in core temperature [65]. Therefore, from a theoretical perspective, a reduction in sweat output owing to the stimulation of abdominal thermoreceptors from drinking cold fluids or ice post-exercise would have less of an impact on heat storage as sweating is rapidly declining anyway. Such an approach may be particularly relevant for participants in intermittent sports with regular breaks. Few studies have empirically investigated this notion; however, Stanley et al. [30] reported a ~ 0.4 °C lower rectal temperature 50-min post-exercise with ice slurry ingestion relative to cold water. Similarly, Lee et al. [29] reported a ~ 0.2 to 0.3 °C greater decline in core temperature with post-exercise ingestion of 4 °C water compared with 28 °C water.

#### Clothing

The boundary environmental conditions presented in Fig. 5a, b are for athletes un-encapsulated by protective equipment/clothing and thus able to fully modify skin surface heat loss using physiological heat loss responses. For more encapsulated individuals, skin surface heat loss is less modifiable as only a small proportion of secreted sweat is able to evaporate, with much of the secreted sweat remaining pooled within the clothing microenvironment. Athletes competing in sports such as American Football, motorcar racing and fencing are regularly exposed to such scenarios, and cold water or ice slurry ingestion by these individuals will most likely create a large net cooling effect because any reductions in sweating will not greatly impact skin surface evaporation.

While no controlled studies to our knowledge have examined the influence of ingested water temperature/ice in encapsulated athletes, studies examining fire fighters also represent a good occupational surrogate population [66]. In that vein, Pryor and colleagues [67] observed an ~ 0.5 °C lower core temperature in fire-fighters ingesting 7.5 g/kg total mass of ice slurry mixture prior to exercise in the heat in a complete fire-fighting ensemble, with core temperature remaining lower relative to 20 °C water ingestion for the first 30 min of exercise. However, more controlled research studies are required to examine this notion further in encapsulated athletes.

#### Physiological Disruptions to Sweating

From a theoretical perspective, cold fluid or ice slurry ingestion should also be beneficial in populations with a physiological disruption to sweating, such as athletes with a spinal cord injury [68, 69], burn injuries [70–72] or following a sympathectomy procedure [73]. In all situations, the maximum proportion of the skin surface that can be physiologically saturated with sweat (i.e. maximum skin wettedness, *ω*_max_) and the associated maximum level of evaporative heat loss are drastically lowered. The decrease in sweating that accompanies the increase in internal heat loss with a cold/ice drink would therefore have a much smaller impact on heat dissipation and yield an improved net cooling effect compared with an athlete with a completely intact sweating apparatus. While the pre-exercise ingestion of ice slurries has been proven to be moderately effective in reducing core temperature prior to the onset of exercise in wheelchair athletes [68], to the best of our knowledge no existing studies have examined the influence of fluid temperature during exercise in the heat on the development of exercise-induced hyperthermia in these populations.

## Note on Different Methods for Estimating Changes in Body Heat Storage During Exercise

Throughout the present review, we have described and discussed the mutual trade-off between the increase in internal heat transfer with an ingested cold fluid and the parallel reduction in evaporative potential from the skin surface secondary to reductions in sweating—most likely modulated by independent thermoreceptors in the abdominal cavity [55, 59]. These two heat transfer components have been estimated using well-established [2, 74] fundamental biophysical relationships (partitional calorimetry), and given that concomitant alterations in dry heat transfer via skin surface convection are minimal with cold fluid ingestion [53], they ultimately determine net body heat storage. However, these calculations may in some cases not align with body heat storage values estimated using the thermometric method in some of the studies reported in our review [14, 15, 18, 19, 23, 25, 26]. For example, Lee and Shirreffs [25] estimated body heat storage to be 33 kJ lower with 10 °C ingestion compared with 50 °C, yet our estimated difference between the increase in internal heat loss and decrease in skin surface evaporative potential for the same study was 40 kJ for the 10 °C fluid and 92 kJ for the 50 °C fluid, indicating a 52 kJ greater potential heat loss with 50 °C fluid.

The thermometric method for estimating body heat storage (which employs a weighted average of the changes in core and skin temperature together with the mean specific heat capacity of the body’s tissues) and total body mass has traditionally been used [75] in many studies captured in this review. However, a series of relatively recent studies has clearly demonstrated, using a reference measure of changes in body heat storage from whole-body direct calorimetry, that values derived using the conventional thermometric approach systematically underestimate the true change in body heat content [76, 77]. Moreover, this systematic error in heat storage estimation via thermometry is magnified with cold water ingestion to the extent that opposite conclusions can be drawn depending on the method employed [50].

## Conclusions and Recommendations

Cold water and ice slurry ingestion during exercise has been demonstrated to exert a positive influence on endurance performance in the heat. Nevertheless, while cold beverages administered during exercise may help an athlete feel cooler, they do not necessarily result in a net cooling effect owing to a reduction in sweating that lowers the potential for evaporative heat loss from the skin by an amount that is at least equivalent to the additional internal heat loss with the ingested fluid. However, when combinations of activity (metabolic heat production) and climate (air temperature, humidity and airflow) conspire to yield decrements in sweating efficiency (i.e. not all secreted sweat evaporates and thus contributes to cooling), a net cooling effect with cold water/ice ingestion develops, as all internal heat loss is always 100% efficient. As such, cold water and ice slurry ingestion can be recommended for cooling an athlete during exercise in hot, humid and still environments; but not warm, dry and windy environments. In other conditions, athletes should ingest fluids at the temperature they find most palatable and therefore best maintain hydration status.

It follows that generally speaking a cold water/ice ingestion would be more beneficial for cooling runners than cyclists mainly by virtue of the differences in airflow across the skin for a given metabolic heat production and concomitant evaporative efficiency of sweat. Cold water/ice ingestion should theoretically be more beneficial for athletes with physiological disruptions to sweating capacity, such as spinal cord-injured athletes, as well as athletes wearing equipment/clothing that present a high level of evaporative resistance, e.g. racing car drivers. However, more research is required in these populations.
